# Cancer-related CD15/FUT4 overexpression decreases benefit to agents targeting EGFR or VEGF acting as a novel RAF-MEK-ERK kinase downstream regulator in metastatic colorectal cancer

**DOI:** 10.1186/s13046-015-0225-7

**Published:** 2015-10-01

**Authors:** Guido Giordano, Antonio Febbraro, Eugenio Tomaselli, Maria Lucia Sarnicola, Pietro Parcesepe, Domenico Parente, Nicola Forte, Alessio Fabozzi, Andrea Remo, Andrea Bonetti, Erminia Manfrin, Somayehsadat Ghasemi, Michele Ceccarelli, Luigi Cerulo, Flavia Bazzoni, Massimo Pancione

**Affiliations:** Medical Oncology Unit, Fatebenefratelli Hospital, 82100 Benevento, Italy; Department of Clinical Pathology, Fatebenefratelli Hospital, 82100 Benevento, Italy; National Institute of Molecular Genetics “Romeo and Enrica Invernizzi”, Milan, Italy; Department of Pathology and Diagnostics, University of Verona, 31134 Verona, Italy; Department of Pathology and Oncology, “Mater Salutis” Hospital, 37045 Legnago, Verona Italy; Bioinformatics Laboratory, BIOGEM scrl, Ariano Irpino, Avellino, Italy; Department of Sciences and Technologies, University of Sannio, Via Port’Arsa, 1182100 Benevento, Italy

**Keywords:** CD15/FUT4, EGFR or VEGF, ERBB3 or FGFR4, Metastatic CRC

## Abstract

**Background:**

Cancer-related immune antigens in the tumor microenvironment could represent an obstacle to agents targeting EGFR “cetuximab” or VEGF “bevacizumab” in metastatic colorectal cancer (mCRC) patients.

**Methods:**

Infiltrating immune cells into tumor tissues, cancer-related expression of immune antigens (CD3, CD8, CD68, CD73, MPO, CD15/FUT4) from 102 mCRC patients receiving first-line Cetuximab or Bevacizumab plus chemotherapy were assessed by immunohistochemistry and validated in an independent tissue microarrays of 140 patients. Genome-wide expression profiles from 436 patients and 60 colon cancer cell lines were investigated using bioinformatics analysis. *In vitro* kinase assays of target genes activated by chemokines or growth factors were performed.

**Results:**

Here, we report that cancer-related CD15/FUT4 is overexpressed in most of mCRCs patients (43 %) and associates with lower intratumoral CD3+ and CD8+ T cells, higher systemic inflammation (NLR at diagnosis >5) and poorer outcomes, in terms of response and progression-free survival than those CD15/FUT4-low or negative ones (adjusted hazard ratio (HR) = 2.92; 95 % CI = 1.86–4.41; *P* < 0.001). Overexpression of *CD15/FUT4* is induced through RAF-MEK-ERK kinase cascade, suppressed by MEK inhibitors and exhibits a close connection with constitutive oncogenic signalling pathways that respond to *ERBB3* or *FGFR4* activation (*P* < 0.001). *CD15/FUT4-*high expressing colon cancer cells with primary resistance to cetuximab or bevacizumab are significantly more sensitive to MEK inhibitors than CD15/FUT4-low counterparts.

**Conclusion:**

Cancer-related CD15/FUT4 overexpression participates in cetuximab or bevacizumab mechanisms of resistance in mCRC patients. CD15/FUT4 as a potential target of the antitumor immune response requires further evaluation in clinical studies.

**Electronic supplementary material:**

The online version of this article (doi:10.1186/s13046-015-0225-7) contains supplementary material, which is available to authorized users.

## Introduction

In the last decade, metastatic colorectal carcinoma (mCRC) treatment has radically improved, thanks to the introduction, into clinical practice of novel agents targeting EGFR and VEGF pathways [1–5].

Cancer is driven by activating mutations and aberrant signal transduction node, most of which (RAS, PTEN, EGFR) play a significant role in the prediction of resistance to epidermal growth factor receptor (EGFR) monoclonal antibodies in mCRC treatment, whereas, equivalent reliable predictors of bevacizumab are currently lacking [1, 2, 6, 7]. Current data provide evidence for extensive autocrine and paracrine EGFR-VEGF (R) cross-talk in both tumor and tumor-associated microenvironment underlining the potential interest in targeting both pathways [1–4]. Although clinically distinct subtypes of CRC are starting to emerge, the factors determining whether a patient will have a response to target-oriented therapy remain still elusive [7, 8].

A tumor grows in an intricate network of epithelial cells, vascular and lymphatic vessels, cytokines and chemokines, and infiltrating immune cells named the tumor microenvironment [9–11]. Increasing studies underscore the involvement of immune cells, through an emerging hallmark of cancer, evoked as evasion of immune surveillance [9–11]. In keeping with this concept, a higher infiltration of memory cytotoxic Th1 T-lymphocytes and tumor-associated neutrophils (TANs) correlate with a longer survival, evidencing the critical effect of host immune response on tumor evolution and clinical outcome [11–16]. Unfortunately, tumor develops multiple mechanisms of evading immune responses, by forming a compromised microenvironment that contrasts the effects of therapeutic agents [10, 17–19]. We have recently proposed that malignant cells can “express” ectopic immune epitopes, which are typical of immune cells (i.e., CD73, CD68) and that these may serve as tumor antigens to evade immune surveillance facilitating homotypic interactions in distant organs during metastatic process [20]. The connection between immune neoantigens expressed on tumor cells and benefits to target therapies has received little attention so far. In this study, we investigated the relationship between inflammatory response, tumor immune-phenotypic features and patients’ outcome receiving first line cetuximab or bevacizumab plus chemotherapy schedules. Herein, we provide evidence that the “don’t eat me” signal CD15/FUT4 on cancer cells associates with decreased benefit to target therapy. CD15/FUT4 overexpression is driven by constitutive oncogenic signalling pathways in the tumor cells (innate immune resistance) acting as a novel RAF-MEK-ERK kinase downstream regulator through *ERBB3 or FGFR4* activation, respectively. The results presented here could help to identify a subset of CD15/FUT4-overexpressing patients who have higher chances of benefiting from MEK inhibitors.

## Patients and methods

### Patient population and samples

To study the relationship between tumor-associated immune infiltration and responses to targeted therapies, between 2010–2014 a retrospective cohort of metastatic CRC patients from two institutions: Medical Oncology Unit of Sacro Cuore di Gesù, Fatebenefratelli Hospital, Benevento (Italy) and Department of Oncology and Pathology, Mater Salutis Hospital, Legnago Verona, (Italy) were recruited. The cohort was partitioned into a discovery and validation set, resulting in a total of (*n* = 102) patients receiving the target-agents Cetuximab or Bevacizumab plus chemotherapy based schedules (FOLFOX, XELOX or FOLFIRI) first-line therapy. Ethical, legal, and social implications were approved by an ethical review board of Fatebefratelli Hospital Institution. Formalin-fixed, paraffin-embedded (FFPE) tumor tissues were retrieved, anonymized and areas of tumor revaluated on hematoxylin and eosin–stained sections. For systemic inflammatory response, the neutrophil-to-lymphocyte ratio (NLR) was calculated from routine complete blood on the same day of primary surgery. Study endpoints were represented by evaluation of patients’ outcome in terms of objective response (primary endpoint), progression free survival (PFS) and overall survival (OS). The best overall response was evaluated every 8 or 12 weeks. Tumor response was classified in: complete response (CR), partial response (PR), stable disease (SD), and progressive disease (PD) according to Response Evaluation Criteria in Solid Tumors (RECIST). Consequently, patients with CR, PR and SD ≥ 6 months were considered responders while the remaining nonresponders. Details of patients’ outcome evaluation are provided in (Additional file 1: Table S1 and Additional file 2).

An independent cohort, named as validation series II consisting of 140 stage I-IV primary CRCs collected consecutively was then evaluated [21, 22]. They represented a continuous, unselected TMAs cohort of patients with molecular, histopathological and clinical findings (OS) recruited during the period 2003–2009. The NLR ratio was collected on the same day of primary surgery for routine laboratory analysis through the full blood count as indicated above. The complete workflow of the study is summarized in (Additional file 3: Figure S1). Details about this data set are provided in (Additional file 1: Table S2 and Additional file 2).

### Immunohistochemistry

Following pathologic review for diagnostic confirmation and exclusion of highly fibrotic or necrotic tumor sections, whole-blocks 4-μm sections were incubated with antibodies listed in (Additional file 1: Table S3). The 3,3’ diaminobenzidine and haematoxylin were used as chromogen and counterstain, respectively. Tumor-associated infiltrating immune cells and cancer-related expression were identified using staining positivity analysis. Infiltrating immune cells from different tumor locations, were quantified by using ImageJ-based software, while intraepithelial immune cells were counted manually. All the cell counts were expressed as cells mm^−2^. The data were obtained from two whole sections per tissue and at least 500 epithelial cells and 500 infiltrating immune cells were analyzed per section. The proportion of cancer cells staining was scored in 3 s grades regardless of intensity as follows: 1) Negative staining (Neg) was defined as the complete absence of staining in more than 95 % of tumor cells; 2) Partially positive or (low expression) characterized by a limited number of tumor cells scattered in a background of either negative or positive tumor cells. 3) Diffuse positivity (High expression) corresponding to a homogeneous membrane staining in virtually all tumor cells. Details on immunohistochemistry (IHC) evaluation is provided in (Additional file 2).

### Bioinformatics analysis and independent gene expression profile data sets

The following genome-wide expression data sets were analyzed by using *in silico* bioinformatics approaches: a) GSE17536/GSE17537 of 226 patients; b) colorectal Cancer Genome Atlas (TCGA) of 210 patients; c) Cancer Cell Line Encyclopedia, Broad Institute/Novartis of 60 CRC cell lines: d) metastatic CRC cell line “SW480” with primary resistance to cetuximab and treated with MEK inhibitor (AZD6244, Selumetinib), GEO Omnibus [7, 23–25]. The IC50, a direct indicator of drug efficacy, for six CRC cell lines, CD15/FUT4-high (HT29, LoVo, SW620) and CD15/FUT4-low (SW480, HCT116, SW48 and GEO) treated with MEKi BAY 86–9766, Selumetinib or Pimasertib was publically available and calculated according to the reported data [26]. Details about *in silico* analysis is provided in (Additional file 2).

### CRC derived cell lines and qRT-PCR validation

A series of 12 representative CRC-derived cell lines, “purchased from American Type Culture Collection (ATCC, Rockville, MD)” were grown in DMEM (Life Technologies, Grand Island, NY, USA) or RPMI 1640 medium plus 10 % FBS (Life Technologies) without antibiotics/antimycotics. All the cell lines were confirmed to be negative for mycoplasma by PCR (Venor GeMkit,Sigma-Aldrich, St. Louis, MO, USA) prior to use. Cells were cultured in a humidified 37 °C incubator at 5 % CO2. Total RNA from cell lines was extracted using miReasy kit (Qiagen, Hombrechtikon, Switzerland) and cDNA was generated using Superscript reverse transcriptase (Life Technologies, Grand Island, NY, USA). The concentration of cDNA was determined (Nanodrop 2000, Thermo Scientific, Asheville, NC, USA) and 25 ng of total cDNA was subjected to quantitative PCR using QI Agility (automated PCR setup, Qiagen), Quanti Tect SYBR Green PCR kit (Qiagen), and Rotor-Gene Q (Qiagen) real-time PCR machine and gene specific primers (Additional file 1: Table S4). The gene-specific copy number was calculated according to the standard curve and normalized to the amount of cDNA (ng) in the reaction. All PCR reactions were performed in triplicate and expression levels were computed as reported [20, 21, 27].

### Reagents, transcript induction and kinase assays

CRC cells were then grown to 70 % of confluence, serum starved for 24 h, and stimulated for 8 h with 10 nM EGF (R&D System), 20U/ml IL-1beta (Peprotech), or for 30 min with 200U/ml IL-10 or 50 ng/ml IL-6 (R&D System). Subsequently, the cells were harvested for RNA (qRT-PCR see above) or protein extraction. Western blot was performed according to the published procedures [20, 21, 27]. A ratio of normalized ERK1/2 (pERK/total ERK1/2), Stat3 (pStat3/total Stat3) and stat1 (pstat1/total Stat1) was calculated for monitoring expression and phosphorylation levels. Human polymorphonuclear cells (PMN) and peripheral blood mononuclear cells (PBMC) purified from buffy coats of healthy donors were used as positive control for kinase assays [27]. Details on western-blot and kinase assays are provided in (Additional file 2).

### Statistical analysis

Statistical analyses were conducted by using R statistical software and SPSS version 15 Windows, SPSS Inc, Chicago, IL and GraphPad Prism 5. Data are presented with medians and ranges. Association between IHC expression and clinico-pathological data was assessed using Spearman r correlation or *χ*2 test. The Wilcoxon–Mann–Whitney or Kruskal-Wallis nonparametric tests were used to identify markers with a significantly differentexpression among patient groups. Kaplan-Meier curves were used to visualize differences between PFS and OS. Significance among patient groups was calculated by using the log-rank test. Prognostic and predictive effects were assessed using PFS and OS as clinical endpoints. The predictive performance of each individual marker was considered alone and together (CD15/FUT4 IHC and NLR at diagnosis). We used Cox proportional hazardsmodel to determine hazard ratios (HRs) with 95 % of confidence interval. The HRs were corrected through multivariate analysis, adjusting for other factors previously shown to be prognostic in the population study. All tests were two-sided, and a *P* < .05 was considered statistically significant. The (Additional file 2) illustrates further details about the statistical and validation methods used.

## Results

### Relationship among inflammatory cell infiltration, immune-phenotypic traits expressed by cancer cells and therapy response

**Fig. 1:**
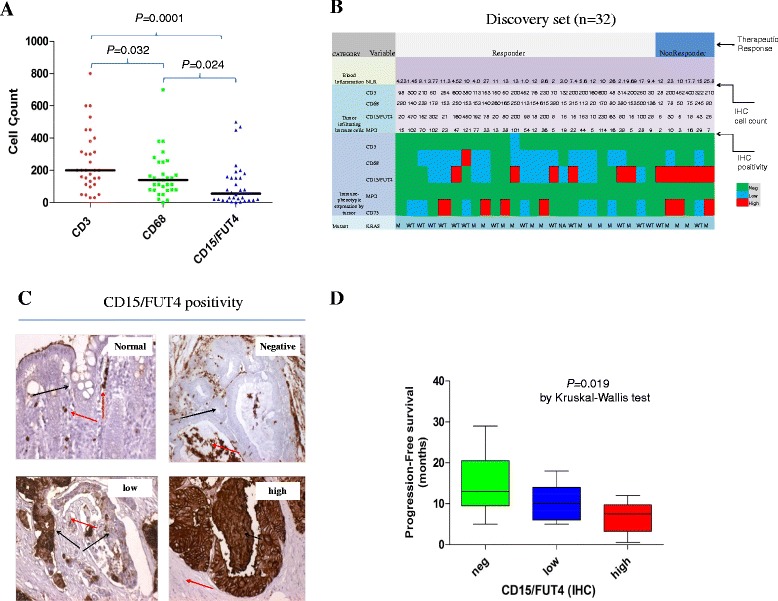
Tumor-associated inflammatory infiltrate, systemic inflammatory response and therapeutic response in the discovery set of mCRC. **a** Tumor-associated quantification of individual cell types, CD3^+^ T cells, CD68^+^ monocyte-macrophage cells, CD15/FUT4^+^ neutrophils (*n* = 32) considering all tumor locations (invasive front, stroma and intraepithelial). One dot represents mean of five replicates cells counts, expressed as cells mm^−2^. Bars represent medians (200, 140 and 56.5 cells counts, respectively). **b** Clustering analysis according to the patients’ best response to first line therapy. Interrelationship among, quantification of four types of inflammatory cells, systemic inflammatory response (NLR at time of diagnosis), tumor-related expression of immune markers including (CD73) and KRAS mutational status in each patient of the discovery set. Tumor infiltrating immune-cells include an additional one, MPO^+^ for neutrophils granulocytes). Immune-positivity on malignant cells is expressed as negative (green), low (blue) and high (red) (see methods for score details). Of note, all patients progressing to first line therapy overexpressed CD15/FUT4 on cancer cells. **c** Representative pictures for CD15/FUT4 IHC in normal mucosae and tumor specimens, respectively. CD15/FUT4 marks intensely neutrophils granulocytes (red arrows) but not or weakly colonic epithelial cells in normal mucosa (black arrow). Cancer-related CD15/FUT4 expression pattern detected in tumors: red arrows indicates stromal compartment, black arrows indicate malignant cells. **d** Relationship between cancer-relatedCD15/FUT4 expression pattern (negative, low and high subgroups) and Progression-Free Survival (PFS) after first line therapy. Distribution of PFS expressed in months, into subgroup CD15/FUT4-high, low and negative tumors, respectively. Bars represent medians (13, 11 and 7.5 months, respectively). Abbreviations: neutrophil-to-lymphocyte ratio, NLR

In the initial discovery set, peritumoral inflammatory infiltrate was, in general, heavier than intratumoral and stromal infiltrate. The CD3+ T cells were the most abundant in all tumor locations (at the invasive front, stroma and intraepithelial), followed by infiltrating CD68+ monocyte–macrophage and CD15/FUT4+ neutrophils, respectively (Fig. 1a).

Next, to distinguish between inflammatory infiltrate and tumor immune-phenotypic traits, we examined the presence of positivity in malignant cells including a larger series of immune markers (Fig. 1b and Additional file 3: Figure S2A,B). Overall, we observed that the neutrophil antigen CD15/FUT4 was positive in the majority of cases. Among the 32 tumors of the discovery set, 23 (72 %) of CD15/FUT4-positive tumors were classified as low or high and had a higher recurrence than other tumor-related immune antigen i.e., CD68, CD73 (Fig. 1b, c and Additional file 2: Figure S2A). Using myeloperoxidase (MPO) as an additional marker of mature neutrophils, we did not detect positivity on malignant cells. In line with this, CD15/FUT4 marked infiltrating granulocytes but not epithelial colonic cells in matched normal adjacent mucosa (Fig. 1c and Additional file 3: Figure S2A, B). Tumors displaying high CD15/FUT4 expression showed a trend towards: a) lower peritumoral immune-cells density and elevated NLR at diagnosis; b) poorer patients’ outcome both in terms of response to first line therapy and progression free survival (Fig. 1b-d and Additional file 3: Figure S2C, D).

### Prognostic significance of tumor-related CD15/FUT4 overexpression and inflammatory response

To corroborate CD15/FUT4 cancer-related alteration and its relationship with inflammatory response and patients’ outcome, we analyzed 70 additional samples, resulting in a total of 102 patients. Additional file 1: Table S1 summarizes clinico-pathological features of the entire study cohort.

**Fig 2:**
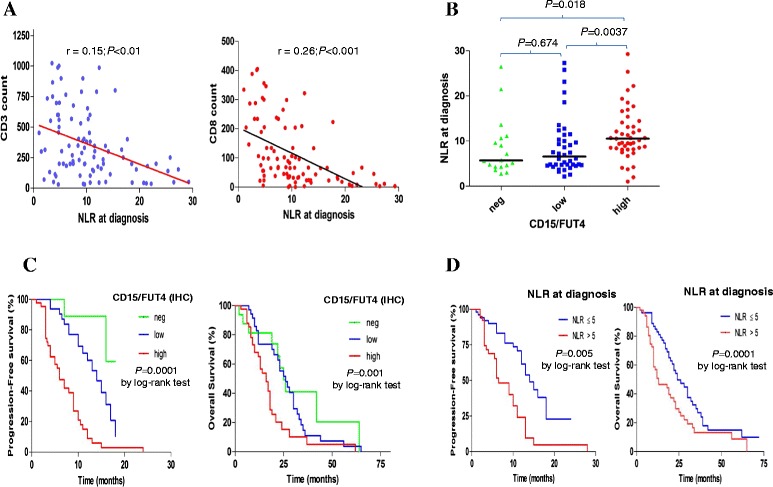
CD15/FUT4 expression pattern, inflammatory response and patients’ outcome in a larger cohort study. **a** Significant inverse correlation of NLR at diagnosis with tumor-associated CD3^+^ T-cells and CD8^+^ T- cytotoxic quantification (*n* = 99; expressed as mean of replicates cells counts, cells mm^−2^). **b** Correlation between NLR at diagnosis and tumor-related expression pattern of CD15/FUT4 spared into three subgroups negative, low and high, respectively. One dot represents NLR at diagnosis for each patient, *P* value was obtained by Mann–Whitney test. **c** Kaplan–Meier curves for progression-free survival and overall survival in the validation set (*n* = 102). The medians PFS value were 13, 10 and 5.5 months (HR = 3.37; 95 % CI = 2.14–5.51). The medians OS value were 38, 26 and 13 months for negative (*n* = 18), low (*n* = 40) and high-CD15/FUT4 (*n* = 44) expressing tumors, respectively (HR = 1.95; 95 % CI = 1.37–2.98). **d** Kaplan–Meier curves for progression-free survival and overall survival in relationship with NLR at diagnosis (cutoff value of 5). The medians PFS value were 12 and 6.5 months respectively (HR = 2.41; 95 % CI = 1.37–4.32). The medians OS value were 35 and 17 months with an NLR ≤5 (*n* = 30) and an NLR >5 (*n* = 72) patients respectively(HR = 2.39; 95 % CI = 1.48–3.85)

Linear correlation analysis showed that high systemic inflammation is associated with lower immune-cells density, especially CD3^+^T cells (r =0.15; *P* < 0.01). A finding further supported by decreased infiltration of CD8^+^ for cytotoxic T cells (r = 0.26; *P* < 0.001; Fig. 2a and Additional file 3: Figure S2C, D).

CD15/FUT4 expression had a significant direct correlation with systemic inflammatory response at diagnosis. The median values of NLR ranged from 5.71 for CD15/FUT4-neg, 6.60 for CD15/FUT4-low and 10.5 for CD15/FUT4-high expressing tumors, respectively (*P* < 0.05; Fig. 2b and Additional file 3: Figure S3A). This indicated that when CD15/FUT4 increases in malignant cells, CRC inflammatory infiltrate declines and systemic inflammation at diagnosis increases.

We then associated cancer-related expression of CD15/FUT4 with patients’ outcome in terms of Response and Survival (PFS and OS).

Among 102 assessable tumor specimens, 31 out of 44, (70 %), 8 out of 40 (20 %) and 1 out of 18 (6 %) of the CD15/FUT4-high, low and negative tumors were considered nonresponders to the therapy, respectively (*P* = 0.00000532; Table 1).

**Table 1:** Response to treatment according to CD15/FUT4-IHC on primary tumors and NLR at diagnosis

	CD15/FUT4-IHC							NLR at diagnosis				
Therapeutic response	Neg		Low		High		*P*	NLR ≤5		NLR >5		*P*
Frequency	N	(%)	N	(%)	N	(%)	0.00000532	N	(%)	N	(%)	0.00012545
Responder	17	(94)	32	(80)	13	(30)		28	(93)	34	(47)	
Nonresponder	1	(6)	8	(20)	31	(70)		2	(7)	38	(53)	
Total	18	(100)	40	(100)	44	(100)		30	(100)	72	(100)	

*Abbreviation*: *IHC* immunohistochemistry, *NLR* blood neutrophil-to-lymphocyte ratio

PFS was significantly different according to CD15/FUT4 expression on malignant cells: mPFS was 5.5 vs 10 and 13 months, in patients with CD15/FUT4-high, low and negative tumors, respectively (HR = 3.37; 95 % CI = 2.14–5.51; *P* < 0.0001, Fig. 2c). Accordingly, median OS was 13 vs 26 and 38 months in patients with CD15/FUT4-high, low and negative tumors, respectively (HR = 1.95; 95 % CI = 1.37-2.98; *P* =0.001, Fig. 2c).

Concordance for systemic inflammatory response at time of diagnosis and clinical response was also significant (Additional file 3: Figure S3B). Thirty-eight out of 72 (53 %) with NLR >5 and 2 out of 30 (7 %) patients with NLR ≤ 5 were considered nonresponders, respectively (*P* **=** 0.00012545; Table 1). Median PFS was 6.5 vs 12 months in patients with NLR >5 vs ≤ 5, respectively (HR = 2.41; 95 % CI = 1.37–4.32; *P* = 0.002). Median mOS was 17 vs 35 months in patients with NLR >5 vs ≤ 5, respectively (HR = 2.39; 95 % CI = 1.48–3.85; *P* < 0.0001; Fig. 2d).

Multivariate Cox regression analysis revealed that systemic inflammation at diagnosis and KRAS mutation were independent prognostic factors for OS, whereas CD15/FUT4, and marginally systemic inflammatory response were independent predictors of PFS after adjustment for therapeutic regime (Table 2). The stratification of patients who presented both positive characteristics revealed that a large proportion (32 out of 40; 80 % of patients) among the nonresponder subgroup had the combination CD15/FUT4^high^/NLR^>5^(Additional file 3: Figure S3C).

**Table 2:** Univariate and multivariate analysis for PFS and OS. Cox’s regression model taking into account the IHC data or NLR at diagnosis

Variable	Univariate (OS)	*P* value	Multivariate (OS)	*P* value
NLR >5 vs NLR ≤5	2.39 (1.48–3.85)	0.0001**	1.99 (1.2–3.3)	0.007**
RAS Mut vs WT	2.07 (1.28–3.34)	0.003 **	1.88 (1.16–3.06)	0.01*
Multiple vs single metastases	1.34 (0.99–1.81)	0.056	1.30 (0.91–1.86)	0.13
CD15/FUT4 high vs low/neg	1.95 (1.37–2.98)	0.001**	1.55 (1.06–2.89)	0.056
Variable	Univariate (PFS)	*P* value	Multivariate (PFS)	*P* value
NLR >5 vs NLR ≤ 5	2.41 (1.37–4.22)	0.002**	2.47 (1.4–4.34)	0.05*
Stage at diagnosis III and IV vs II	1.42 (1.05–1.91)	0.021*	1.3 (0.928–1.81)	0.127
Histotype mucinous vs others	1.60 (1.0–2.57)	0.05*	1.43 (0.89–2.32)	0.136
CD15/FUT4 high vs low/neg	3.37 (2.14–5.51)	0.0001**	2.92 (1.86–4.61)	0.001**

*Abbreviations*: *HR* Hazard ratio, *95 % CI* 95 % confidence interval, *IHC* Immunohistochemistry, *NLR* blood neutrophil-to-lymphocyte ratio

*P ≤ .05; **P ≤ .01

### Tumor-related CD15/FUT4 expression and associated molecular parameters in an independent TMAs validation set of stages I-IV CRCs

This independent cohort confirmed CD15/FUT4 positivity in the majority of carcinomas (76 %; 107 out of 140). High, low and negative expression was identified in 58 out 107 (54 %), 49 out of 107 (46 %), and 33 out of 107 (24 %) of cases, respectively (Additional file 1: Table S2 and Additional file 3: Figure S4A). Tumors displaying CD15/FUT4-high expression pattern were more frequently associated with advanced tumor stage III and IV and with lower-densities of CD3+ and CD8+ T cells peritumoral infiltrate, confirming the data obtained on the first series (Additional file 3: Figure S4B). Tumors displaying this pattern were also frequently MMR proficient and TP53 and KRAS mutated than CD15/FUT4-negative one (Additional file 1: Table S3). We tested whether the combination CD15/FUT4 and NLR had impact on patients’ prognosis also in this series. Indeed, the combinations CD15^Neg^NLR^≤5^ and CD15^high^NLR^>5^robustly stratify the cohort into two groups with 100 % and 33.1 % of survival rate at 5 years after diagnosis, respectively. (Additional file 3: Figure S4C, D).

### Genomic variations related to *CD15/FUT4* overexpression

**Fig. 3:**
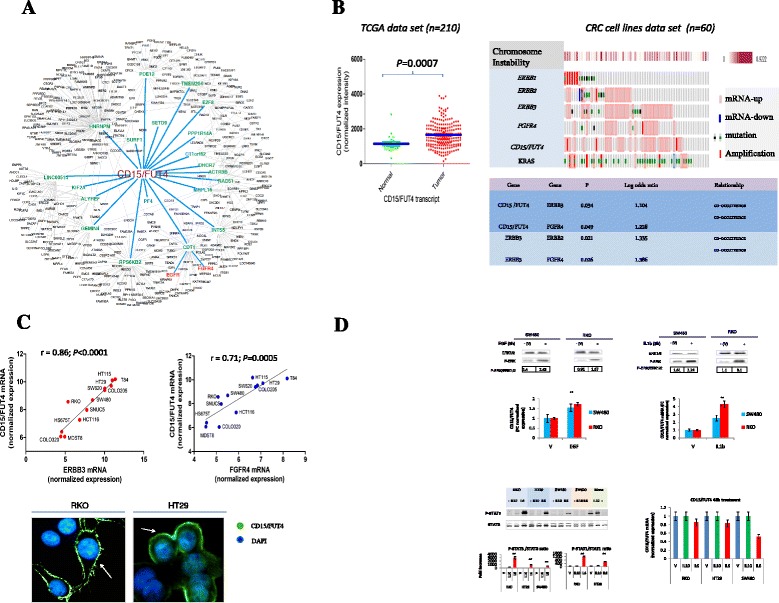
Genome-wide expression analysis identifies CD15/FUT4 as a novel RAF-MEK-ERK kinase downstream target. **a** The most enriched network of *CD15/FUT4* regulators is depicted comprising 20 regulons and two closely connected upstream genes involving EGFR and FGFR pathways (see Additional file 2 and Additional file 3: Figure S5 for more information). The gene expression datasets GSE17536/GSE17537 series (*n* = 226) were analyzed simultaneously with the ARACNe algorithm to infer transcriptional regulatory network fromgenome-wide expression profiles CD15/FUT4-connected. **b** *CD15/FUT4* expression in CRC using patient-matched tumor-normal data available from TCGA. The *P* value refers to Mann–Whitney test. Expression profiles of *ERBB1, ERBB2, ERBB3, FGFR4, CD15/FUT4* and KRAS mutant across a series of CRC cell lines (*n* = 60) by applying a fold change of gene expression microarray data of 1.5 and subdivided on the basis of chromosomal instability calculated as fraction of copy-number alteration pattern (form 0 to 0.922). Significant correlation between *CD15/FUT4,* ERBB3 and FGFR4 expression levels. **c** Linear correlation between *ERBB3, FGFR4* and *CD15/FUT4* transcript levels is validated in our independent series of (*n* = 12) CRC cell lines. Immunofluorescence labeling “green” of nonpermeabilize cancer cells shows differential expression of CD15/FUT4 “arrows,” on the cell plasma membrane of RKO and HT29, respectively. To visualize nuclei, the merged images were stained with 4’6-diamidino-2-phenylindole (Dapi), “blue”. **d** CD15/FUT4-dependent transcript induction by EGF (10 nM) or IL1b (20U/ml) by using 0.1 % of DMSO as vehicle, in two representative CRC cell lines SW480 and RKO, respectively. Cells were treated for 8 h and the ratio anti-p-ERK/ERK1/2 was quantified by western-blot analysis. The treatment with IL-10 (200U/ml) or IL-6 (50 ng/ml) for 30 min revealed a significant induction of IL6-STAT1/STAT3 dependent phosphorylation in a panel of 4 CRC cell lines as detected by western-blot analysis. Mononuclear cells (Mono) purified from buffy coats of healthy donors were used as positive control for IL10-STAT3 dependent phosphorylation. *CD15/FUT4* transcript did not reveal any significant induction following IL10 or IL6 at 48 h of treatment. The *P* value were obtained by Mann–Whitney test; *P ≤ .05; **P ≤ .01

To gain insights into the mechanisms that regulate *CD15/FUT4* expression, we collected two independent publicly available gene-expression datasets involving a total of 436 colorectal cancer and 60 colon cancer cell lines [7, 23, 24]. Strikingly, *in-silico* analysis, inferred a number of 1350 interactions and 20 regulons *CD15/FUT4-*associated, and revealed a series of disease-relevant pathways: a) prosurvival; b) immune-evasion; c) protein kinase cascade and viral infectious response closely connected to the efficacy of drugs (Fig. 3a and Additional file 3: Figure S5A-D) [28–30]. *CD15/FUT4* transcript was significantly higher in tumor tissues than normal control in TCGA series comprising 210 CRCs and 22 and normal colorectal mucosa specimens, respectively (Fig. 3b). We further confirmed the association between *CD15/FUT4* overexpression and cancer recurrence in the GSE17536/GSE17537 series (*n* = 226), for which reported follow-up data were available (Additional file 3: Figure S6A, B) [23]. In this cohort, *CD15/FUT4* overexpression was associated with short time-to-recurrence (TTR) but not with OS, independently from initial tumor stage (Additional file 3: Figure S6B). *CD15/FUT4* transcripts were significantly higher in chromosomal instability (CIN) positive than CIN-negative tumors. Its upregulation was associated to relevant genetic aberrations such as: *ERBB2*, *ERBB3* and *FGFR4* overexpression, a finding confirmed in a large series of 60 CRC cell lines (Fig. 3b and Additional file 3: Figure S6A-C).

### CD15/FUT4 overexpression and MEK inhibitor responses in CRC cell lines

**Fig. 4:**
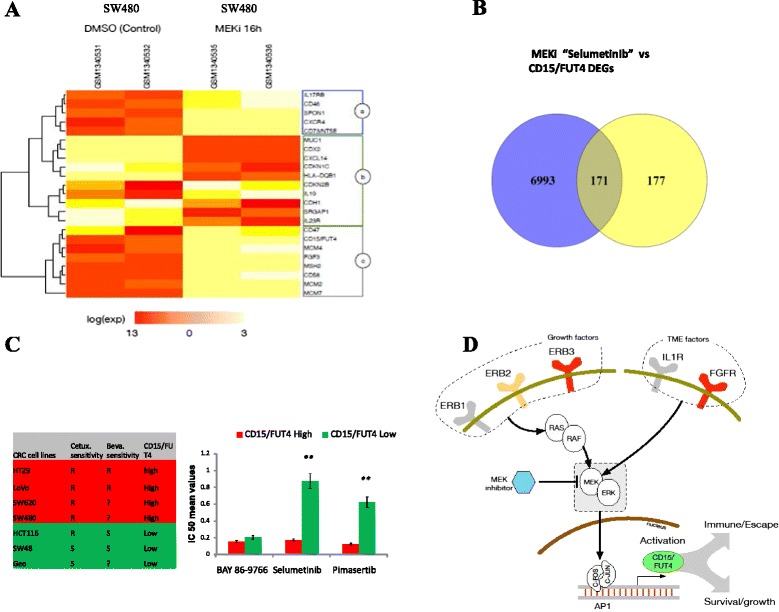
CD15/FUT4 transcript is repressed by MEK inhibitors in CRC cell lines with intrinsic resistance to cetuximab or bevacizumab. **a** Heat map showing groups of differentially expressed genes or “responsive genes” to the MEK inhibitor “AZD6244, Selumetinib” (MEKi) or control, in a metastatic, KRAS mutant and cetuximab-resistant CRC line “SW480”. Down-regulated expression of *CD15/FUT4* in “Selumetinib” treated cells, shows a high level of concordance with genes implicated in DNA replication (MCM) and immune-escape mechanisms (CD47, CD73) (gene signature *a* and *b*, respectively). The gene signature (*b*) indicates MEKi upregulated genes mainly involved in cell-cycle arrest (CDKN) or tumor differentiation (CDX2, CDH1, MUC1). **b** Number and distribution of differentially expressed genes (DEGs) between the MEK inhibitor responsive genes and CD15/FUT4-related genes derived from (TCGA data set). **c** Six CRC cell lines, HT29, LoVo, SW480, SW620, HCT116, SW48 and GEO treated for 96 h with indicated MEKi are subdivided taking into account *CD15/FUT4* expression levels. Results represent the mean of the IC50 ± SD determined by interpolation from the dose response curves in CD15/FUT4-high (HT29, LoVo, SW480 and SW620) and CD15/FUT4-low (HCT116, SW48 and GEO) expressing CRC cells. HT29, LoVo, SW480 and SW620 cells are known for intrinsic resistance (R) to cetuximab or bevacizumab. **d** Simplified model showing CD15/FUT4 activation as a novel RAF-MEK-ERK signaling cascade downstream regulator. CD15/FUT4 could promote two mechanisms of primary resistance to agents targeting EGFR and VEGF cell proliferation (prosurvival) and evasion of immune surveillance likely through several pathways consisting of ERBB3, FGFR and IL1b cascade. The combined blockade of EGFR or VEGF with MEKi could represent a therapeutic strategy for preventing and/or overcoming resistance of CD15/FUT4-overxpressing tumors

We then tested this observation by performing qRT-PCR assays on a panel of CRC cell lines. This analysis revealed that *CD15/FUT4* overexpression is tightly correlated with *ERBB3* and *FGFR4* transcript but not with EGFR. Of note, expression of CD15/FUT4 on the surface of tumor cells indicated a good concordance with transcript levels (Fig. 3c). Based on these observations, to verify whether *CD15/FUT4* could be regulated by kinase cascade, we treated RKO and SW480 CRC cells with EGF or IL1β for 8 h. Following exposure to EGF or IL1β, we observed a consistent induction of *CD15/FUT4* transcript through ERK1/2 phosphorylation (Fig. 3d). In contrast, IL10 or IL6- STAT1/STAT3 dependent phosphorylation did not cause *CD15/FUT4* overexpression (Fig. 3d). To further confirm *CD15/FUT4* as a RAF-MEK-ERK kinases downstream effector, we used two recently published gene expression data sets [25, 26] derived from metastatic CRC cell lines, “with primary resistance to cetuximab” querying for MEK inhibitor (MEKi) responsive genes (Fig. 4a). We found that *CD15/FUT4* transcript was significantly downregulated following “Selumetinib” treatment (FDR < 0.01 related to untreated control) and showed a high level of synergy with gene signature related to the prosurvival and evasion of immune surveillance (Fig. 4a, b). To further demonstrate that MEKi growth inhibition effect was dependent on *CD15/FUT4* expression levels, we used public data from three selective MEK inhibitors, BAY 86–9766, Selumetinib and Pimasertib in a panel of six CRC cell lines. We observed that both “Selumetinib and Pimasertib” were highly effective in *CD15/FUT4*-high expressing CRC cells with intrinsic resistance to cetuximab or bevacizumab, as compared to those with CD15/FUT4-low expression one (Fig. 4c).

## Discussion

Much effort is currently focused on attempts to target several signaling pathways at the same time. The extensive degree of EGFR-VEGF(R) pathway cross-talk identifies them as particularly promising for joint targeting [3, 29]. In addition to *RAS* gene family mutations, a number of studies suggest that intracellular downstream effectors of these pathways or immune inflammatory microenvironment could be correlated with primary resistance in metastatic tumors [1–5, 10]. Tumor microenvironment not only plays a pivotal role during cancer progression and metastasis but also has profound effects on therapeutic efficacy [10–13, 17–19]. According to this assumption, immune checkpoint inhibitors are promising new approaches for tackling solid tumors [31–33].

In this study, we investigated candidate determinants of resistance related to the immune tumor microenvironment and inflammatory response, using as proof of principle two mainstay of first line treatment for metastatic CRC anti-EGFR cetuximab, anti-VEGF bevacizumab based therapy. Surprisingly, among a panel of immune markers, we uncovered that a large proportion of tumors overexpressed CD15/FUT4 neuthrophil antigen. Strikingly, this expression pattern associated with short disease control and rapid disease progression. CD15/FUT4-high expressing tumors showed lower intratumoral immune density of both innate (CD68+, CD15+ and MPO+ macrophages and Neutrophil cells) and especially adaptive (CD3+ and CD8+) immune T-cell subsets. The concordance with NLR at diagnosis, suggested that CD15/FUT4 overexpression on malignant cells could connect peritumoral immune suppression and elevated systemic inflammatory response. Patients with tumors harboring CD15/FUT4-high expression were associated with worse PFS both in univariate and multivariate analysis, indicating CD15/FUT4 as a possible marker of decreased therapy response, a finding further strengthened by an elevated blood inflammatory response (NLR > 5). Therefore, the progressive decrease of immune cell densities along with CD15/FUT4 overexpression and increased inflammatory response could provide a clinical context of tumor progression, explainable as a pronounced immune-escape mechanism. Our observations however have a number of limitations in particular because patients had received two treatments targeting different pathways, and are few to support more general conclusions, although the number can be considered large (102) for an IHC study. In this regard, an independent TMAs data set confirmed that high-CD15/FUT4 expressing tumors correlated with reduced density of the immune infiltrates by CD3 and CD8 cells and elevated blood inflammatory response, similarly to those seen in the validation set I. We observed that CD15/FUT4-expressing tumors had generally a well-differentiated gene expression pattern (MMR proficient) and correlated with subtypes enriched for *TP53* and *KRAS* mutations. These results evidenced that CD15/FUT4 could serve as a surrogate marker to identify specific subtypes with chromosomal instability for which has already been proved lack of response to cetuximab and tendency to metastasize [8]. Despite this, such evidence cannot explain lack of response to agents targeting anti-EGFR and even more to anti-VEGF. In this scenario, we performed *in-silico* analysis across publically available gene-expression data sets along with *in vitro* assays on derived colon cancer cell lines. Transcriptomic profiling confirmed that high *CD15/FUT4* transcript associated with CIN, KRAS mutations and most importantly with *ERBB3* and *FGFR4* overexpression but not *ERBB1* (EGFR). These results supported the hypothesis that consistent activation of *CD15/FUT4* transcript acts downstream and/or independently of EGFR or VEGFR pathways. Interestingly, our findings are also consistent with recent studies revealing that transcriptional induction of ERBB3 acts as a prominent “hit” of intrinsic resistance in CRC cell lines, while very little is known about FGFR4 overexpression [34, 35]. Our observations indicate that upregulation of FGFR4 may correlate with intrinsic resistance to bevacizumab, as evidenced in HT29 and SW620 CRC cell lines, respectively.

Therefore, the novel role of CD15/FUT4 reflects persistent genetic features of tumor cells rather than differences in immune infiltrates. Indeed, we demonstrate *“in vitro”* that *CD15/FUT4* transcript is induced through MAPK-ERK kinase cascade. This finding was further validated by querying for selective MEK inhibitor modulated genes in cancer cell lines with KRAS or BRAF mutations and primary resistance to cetuximab or bevacizumab, respectively. We observed that the MEK inhibitor “Selumetinib” caused a significant reduction of CD15/FUT4 transcript, as a consequence, *CD15/FUT4-*high expressing cancer cells were more sensitive to “Selumetinib” and Pimasertib” than CD15/FUT4-low counterparts.

Based on these findings, we proposed a model where drug resistance could synergize on *CD15/FUT4* through at least two independent pathways: a) mitogenic intracellular due to ERBB2/ERBB3 overexpression; b) pro-angiogenic and/or mitogenic due to FGFR4 or IL-1β released by an altered tumor microenvironment as suggested by other studies (Fig. 4d) [35–37].

Our results suggest a possible rational for treating CD15/FUT4-overexpressing mCRC through means of IHC. In this subset, MEK inhibitors or dual inhibitors that show strong synergy with MEK inhibition “i.e., cetuximab” could be effective for preventing and/or overcoming primary resistance to cetuximab in CRC patients as recently reported in preclinical studies [34, 35]. CD15/FUT4 could also be dysregulated by tumors as an important immune resistance mechanism similarly to immune-checkpoint proteins. However, the relevance of its molecular interactions to inhibit local antitumor T cell-mediated response in the tumor microenvironment remains obscure [38].

In conclusion, cancer-related CD15/FUT4 overexpression is associated with decreased benefit to target and chemotherapeutic agents in metastatic tumors. *CD15/FUT4* acts as a downstream regulator of MAPK-ERK pathway independently of EGFR or VEGF pathway, by coupling mitogenic signaling cascade and immune-escape mechanisms of metastatic tumors. Upregulation of CD15/FUT4 on the tumor cell surface could represent a potential target to enhance antitumor effector functions in the tumor microenvironment. In addition, IHC assessment of CD15/FUT4 combined with RAS mutation status could be a strategy to identify mCRC patients who have higher chances of benefiting from targeting and MEK inhibitor drugs. However, its role as a potential biomarker susceptible to specific therapeutic agents requires further evaluation in clinical setting.

## Additional files

Additional file 1:
**Supplementary Tables S1-S4.** (DOC 121 kb) Additional file 2:
**Supplementary Methods.** (DOCX 37 kb) Additional file 3:
**Supplementary Figures and legends.** (PDF 767 kb)
