# Supplementary material for: Cancer-related CD15/FUT4 overexpression decreases benefit to agents targeting EGFR or VEGF acting as a novel RAF-MEK-ERK kinase downstream regulator in metastatic colorectal cancer
Source: J Exp Clin Cancer Res. 2015 Oct 1;34:108. doi: 10.1186/s13046-015-0225-7 (PMC4590269; doi:10.1186/s13046-015-0225-7)
Supplement: Additional file 1: — Supplementary Tables S1-S4. (DOC 121 kb) [file 13046_2015_225_MOESM1_ESM.doc]

**Table S1 .** Overall characteristics of metastatic CRC patients receiving Cetuximab and Bevacizumab based first line therapy

| **Characteristics** | **No. %** |
| --- | --- |
| **No. of Patients** | **102** |
| **AGE**  Median | 64 |
| **SEX**  Male  Female | 53 52  49 48 |
| **ECOG Performance Status**  0  1  2 | 46 45  35 34.3  21 20.7 |
| **STAGE AT DIAGNOSIS**  II  III  IV | 21 20.5  29 28.5  52 51 |
| **PRIMARY TUMOR SITE**  Colon  Rectum | 74 72.5  28 27.5 |
| **SITE OF METASTASES**  Liver  Lung  Peritoneum  Lymphonodes  Bone  CNS | 83 81.0  35 34.3  22 21.5  16 15.6  6 5.8  2 2 |
| **NUMBER OF METASTATIC SITES**  1  2  >2 | 49 48.0  44 43.0  9 9.0 |
| **K Ras STATUS**  Wild Type  Mutant  Not Available | 41 40.2  50 49.0  11 10.8 |
| **FIRST LINE CHEMOTHERAPY**  Chemo doublet + Bevacizumab  Chemo doublet + Cetuximab | 54 52.9  48 47.1 |

Abbreviations : ECOG PS, Eastern Cooperative Oncology Group performance status;

|  | | **Tumor-related CD15/FUT4 positivity** | | |  |
| --- | --- | --- | --- | --- | --- |
| **Variable** | | **neg** | **low** | **high** | **P value** |
|  | | **N %** | **N %** | **N %** |  |
| **Gender** | Female | 13 39.3 | 19 38.7 | 18 31.0 | 0.622 |
|  | Male | 20 60.6 | 30 61.2 | 40 68.9 |  |
| **Localization** | Right | 15 45.4 | 18 36.7 | 18 31.0 | 0.388 |
|  | Left | 18 54.5 | 31 63.2 | 40 68.9 |  |
| **Stage** | I | 2 6.06 | 5 10.2 | 6 10.3 | 0.007** |
|  | II | 23 69.6 | 27 55.1 | 16 27.5 |  |
|  | III | 3 9.09 | 5 10.2 | 11 18.9 |  |
|  | IV | 5 15.1 | 12 24.4 | 25 43.1 |  |
| **Histology** | ADC | 27 81.8 | 40 81.6 | 49 84.4 | 0.974 |
|  | ADC-muc | 4 12.1 | 7 14.2 | 7 12.0 |  |
|  | ADC-squam | 2 6.06 | 2 4.08 | 2 3.44 |  |
| **CK20** | Neg | 15 45.4 | 15 30.6 | 15 25.8 | 0.151 |
|  | Pos | 18 54.5 | 34 69.3 | 43 74.1 |  |
| **CDX2** | Neg | 7 21.2 | 3 6.12 | 4 6.89 | 0.049* |
|  | Pos | 26 78.7 | 46 93.8 | 54 93.1 |  |
| **MMR** | Neg | 17 51.5 | 45 91.8 | 50 86.2 | <0.0001** |
|  | Pos | 16 48.4 | 4 8.16 | 8 13.7 |  |
| **TP53** | Neg | 16 48.4 | 7 14.2 | 17 29.3 | 0.008** |
|  | Low | 11 33.3 | 18 36.7 | 16 27.5 |  |
|  | High | 6 18.1 | 24 48.9 | 25 43.1 |  |
| **KRAS** | WT | 23 70.0 | 34 69.3 | 30 51.7 | 0.032* |
|  | MUT | 10 30.0 | 15 30.7 | 28 48.3 |  |
| **Total** |  | 33 100 | 49 100 | 58 100 |  |

**Table S2.** Relationship between CD15/FUT4 (IHC) and clinico-pathological features of the TMAs validation series. **Abbreviations:** Right includes: Proximal caecum, ascending and transverse colon. Left includes: Distal descending, sigmoid colon, rectum. ADC adenocarcinoma, ADC-Muc adenocarcinoma with a mucinous component below 50%, or ADC-squam, adenocarcinoma with squamous component below 50%. Mismatch repair-deficient (MMR positive) tumors showed absence of nuclear staining in at least one of following marker: MLH1 or MSH2 or MSH6 or PMS2. Other markers CK20, CDX2 and TP53 were scored regardless of staining intensity as already reported [20].

**Table S3. Primary antibodies employed for immunohistochemical studies.**

| Primary antibodies | incubation time (min) | Manufacture (Clone) | Dilution |
| --- | --- | --- | --- |
| CD3 | 15 | Novocastra  Laboratories (PS1) | 1:200 |
| CD8 | 15 | Novocastra  Laboratories (4B11) | 1:100 |
| MPO | 15 | Ventana (polyclonal) | 1:150 |
| CD15 | 15 | Ventana (MMA) | 1:100 |
| CD68 | 15 | Ventana (KP-1) | 1:100 |
| CD73 | 60 | Abcam (ab115289) | 1:50 |
| CK20 | 15 | Transduction Laboratories (Ks 20.8) | 1:50 |
| TP53 | 15 | Ventana (DO-7) | 1:100 |
| CDX2 | 30 | Novocastra Laboratories ( AMT28) | 1:50 |
| MLH1 | 30 | Novocastra Laboratories ( ES05) | 1:100 |
| MSH2 | 30 | Novocastra Laboratories (25D12) | 1:100 |
| MSH6 | 30 | Novocastra Laboratories (PU29) | 1:100 |

**Table S4:** Primers employed for qRT-PCR studies. 18S RNA and RPL32 were housekeeping genes for human CRC cell lines and isolated PMN.

| Gene | Sense | Antisense |
| --- | --- | --- |
| CD15/FUT4 | | CCG GCG AAG TTA TCA AGG GTT | | --- | | | AAA GGA ACA ACT TTC CCC GA | | --- | |
| ERBB3 | GGGGAGTCTTGCCAGGAG | CATTGGGTGTAGAGAGACTGGAC |
| FGFR4 | ATGGAACTGGTGTGCTCAAGAAGC | TTCACATGTCCTCCGACCAACACA |
| 18S RNA | GATATGCTCATGTGGTGTTG | AATCTTCTTCAGTCGCTCCA |
| RPL32 | CATCTCCTTCTCGGCATCA | AACCCTGTTGTCAATGCCTC |
