# Supplementary material for: Cancer-related CD15/FUT4 overexpression decreases benefit to agents targeting EGFR or VEGF acting as a novel RAF-MEK-ERK kinase downstream regulator in metastatic colorectal cancer
Source: J Exp Clin Cancer Res. 2015 Oct 1;34:108. doi: 10.1186/s13046-015-0225-7 (PMC4590269; doi:10.1186/s13046-015-0225-7)
Supplement: Additional file 2: — Supplementary Methods. (DOCX 37 kb) [file 13046_2015_225_MOESM2_ESM.docx]

**Cancer-related CD15/FUT4 overexpression decreases benefit to agents targeting EGFR or VEGF acting as a novel RAF-MEK-ERK kinase downstream regulator in metastatic colorectal cancer.**

**Guido Giordano^1*^, Antonio Febbraro^1^, Eugenio Tomaselli^2^, Maria Lucia Sarnicola^3^, Pietro Parcesepe^4^, Domenico Parente^2^, Nicola Forte^2^, Alessio Fabozzi^1^, Andrea Remo^5^, Bonetti Andrea^5^, Erminia Manfrin^4^, Somayehsadat Ghasemi^4^, Michele Ceccarelli^6,7^, Luigi Cerulo^6,7^, Flavia Bazzoni^4^ and Massimo Pancione^7*^**

^1^Medical Oncology Unit, Fatebenefratelli Hospital, 82100 Benevento, Italy; ^2^Department of Clinical Pathology, Fatebenefratelli Hospital, 82100 Benevento, Italy; ^3^National Institute of Molecular Genetics *“Romeo and Enrica Invernizzi”,*Milan, Italy. ^4^Department of Surgery and Oncology, University of Verona, 37129 Verona, Italy;^5^Department of Pathology “Mater Salutis” Hospital, 37045 Legnago (Verona), Italy; ^6^Bioinformatics Laboratory, BIOGEM scrl, Ariano Irpino, Avellino, Italy; ^7^Department of Sciences and Technologies, University of Sannio, 82100 Benevento, Italy.

**Supplementary Methods**

**Patient population and Samples**

Clinical records and archival primary tumor FFPEblock of patients treated with target-agents Cetuximab and Bevacizumab based first line therapy at Medical Oncology Unit of SacroCuoreGesù, Fatebenefratelli Hospital in Benevento (Italy), and Department of Oncology and Pathology, Mater Salutis Hospital, Legnago Verona, (Italy) between January 2010 and December 2013,were retrospectively collected. The patients included in the study (n=102), were considered eligible if they had histologically confirmed metastatic colorectal adenocarcinoma, measurable disease, clinical data regarding medical history, treatment and outcomes and finally whole Formalin Fixed Paraffin Embedded (FFPE) paraffin-embedded block of the primary tumors.

Patients were excluded from our evaluation if they had missing information about clinico-pathological features. The study was approved by approved by the Institutional Review Board of the Fatebenefratelli and Legnago Hospital and carried out in accordance with the Declaration of Helsinki under good clinical practice and full ethics committee approval.

To avoid bias election, patients were enrolled consecutively and consisted of two independent cohorts: a) a discovery set (n=32) to explore the potential relationship among peritumoralimmune infiltration, systemic inflammatory response and patients’ outcome; b) validation set (n=70) to assess the prognostic impact of the identified markers in a larger population comprising a total of 102 cases **(Table S1).**Twenty-two normal adjacent mucosa were available and used as control. Tumors were staged using the conventional tumor, node, metastasis (TNM) staging system, 7th edition, 2010 (AJCC, 2010). Patients with conditions known to elicit an acute or chronic systemic inflammatory response were excluded. Blood samples were collectedon the same day of primary surgery for routine laboratory analysis of full blood count, white cell and lymphocyte counts. The neutrophil-to-lymphocyte ratio (NLR), a robust marker of systemic inflammatory response, was determined by dividing the absolute neutrophil count by the absolute lymphocyte count [1]. In order to determine the optimal cutoff point for clinical use, time-dependent receiver operating characteristic (ROC) curves were created for each outcome measure. The optimal NLR value for each outcome at a given time point was identified by optimizing both sensitivity and specificity. NLR data were then dichotomized in two classes (≤5 or >5), respectively. Therapy response was evaluated by clinicians every 8-12 weeks using computed tomography scan or magnetic resonance imaging according to Response Evaluation Criteria in Solid Tumors (RECIST). Patients who achieved a partial (PR) or a complete response (CR) according to RECIST and patients who achieved a disease stabilization that lasted greater than 6 months were considered responders. Patients who showed a progression disease (PD) or disease stabilization that lasted less than 6 months, conversely, were considered as nonresponders. Progression Free Survival (PFS) was defined as the time elapsed between the start of first line chemotherapy and disease progression, treatment discontinuation or death. Overall Survival (OS) was defined as the time elapsed between the start of first line chemotherapy and death.

**KRAS Mutation Analyses**

DNA was extracted from formalin-fixed paraffin-embedded tumor tissue and from tissue recovered from stained slides used to evaluate IHC expression. *KRAS* mutations at codons 12 and 13 in exon 2 were detected using polymerase chain reaction PCR and/or direct sequencing and validated by real-time PCR as described previously [2]. Investigators who performed molecular analyses were blinded to patient characteristics and outcomes.

**Tissue Microarray construction**

Tissue microarrays (TMAs) were constructed from 140 patients as described previously [2,3]. This independent data set comprised primary tumor specimens (stages I-IV) and 60 matched normal mucosa collected at two institutions (see above) during the period 2003-2009. Tumors wereclassified and graded according to the criteria of the TNM andtumor stages I-IV classification systems. For each patient,the date of colon cancer diagnosis, date of last follow up,and vital status at last follow-up (i.e., living or deceased) were recorded. Cores measuring 0.6 mm in diameter were madein triplicate from paraffin blocks comprising tumor tissues or matched normal mucosa and processed using the ATA-27 automated arrayer (Beecher Instruments). TMA blocks were cut to 4-µm sections, deparaffinized, rehydrated in graded alcohol, and stained with haematoxylin & eosin were then reviewed to ensure that from each case were morphologically similar to those of the corresponding whole tissue section and represented cancerous or normal epithelial cells. Finally, after removing the cores containingtoo little tumor sample, the number of lesions that were available for evaluation included 140 CRCs and 60 normal colonic tissues, respectively. Further 4 μ thick sectionswere then cut from each of the master blocks for immunohistochemical (IHC) analyses. A series of molecular parameters of tumor differentiation (CDX2, CK20)mismatch repair proteins (MLH1 or MSH2 or MSH6 or PMS2), TP53 and KRAS status were included following the and classification previously reported **(Table S2).**

NLR was determined by dividing the absolute neutrophil count by the absolute lymphocyte count on the same day of primary surgery. NLR data were then dichotomizedand given a score of ≤5 and >5 according the above proposed threshold. Cancer-associated inflammation, in the form of local and systemic inflammatory responses was evaluated by CD3 and CD8 count in triplicate TMA cores and NLR at the time of diagnosis.

**Immunohistochemistry and evaluation of staining**

All immunohistochemical results were interpreted by 3 independent observers (E.T, A.R, M.P) blinded to clinical data. Sections (4 µm) cut from whole paraffin embedded or TMA blocks were deparaffinised in xylene and rehydratedthrough graded alcohol series. For antigen retrieval, the sections were pretreated with sodium citrate buffer**,** pH 6.0, in a microwave oven for 15 minutes and processed by using VENTANA automated slide stainers based on avidin-biotin complex **(**ABC**)** and labeled streptavidin binding (LSAB) system. After neutralizingendogenous peroxidase activity, the sections were incubated at 37°C with diluted primary antibodies **(Table S3).** The stained sections at X20 and X10 objectives, from different locations (peritumoural, intratumoural stromal and intratumoural epithelial) by using Nikon Eclipse E600 microscope (Nikon, Tokyo, Japan) were photographed. The proportion of the positive inflammatory cells based on their size, shape, and the intensity of positivity was validated by independent investigators blinded to clinical data.The cancer-related positivity was identified by excluding stromal compartment through hematoxylin and eosin staining and using colonic-specific biomarkers, CK20 and CDX2 immunopositivity. Quantification of each marker was compared with quantification on zones of tumors excluding stromal bands and necrotic areas. All the cell counts were expressed as cells mm^-2^. For TMAs thresholds were set to avoid connective tissue, fat, and necrosis. Median was calculated per tumor replicate. Tumor-related expression of immune markers on malignant cells was defined as granular cytoplasmatic or cytoplasmatic and membrane staining pattern.

**Genome-wide and gene expression profiles analyses**

A total of 436 patients from three public CRC data sets were analyzed to establish genome-wide mRNA expression profiles and provide a consistent molecular classification [4,5]. The association of *CD15/FUT4* gene expression profile *(GEP)*with CRC molecular subtypes, clinico-pathological factors and genome-wide DNA alterations was performed by using a two step process. In the first step we identified CD15/FUT4 as a differentially expressed gene and in the second step we associated its expression with a specific signature or subtypes. The GEPs were screened separately by using a fold-change of at least 1.5 (p value <0.05). The GSE17536 and GSE17537 pooled series of 226 patients were analyzed as previously reported. Within such a cohort, 220 patients with documented relapse (distant and/ or loco-regional recurrence)were also avail­able for survival analysis. We examined therelationship between recurrence and other histopathological information such as Dukes’ stage,age, location of tumors (left or right of colon or rectum). We censored those patients whowere alive without tumor recurrence or dead at last contact.

The transcriptional regulation network of this series was inferred using the ARACNE algorithm which adopts the mutual information measure to infer potential interactions between transcription factors and targets [6,7]. Mutual information was estimated by using parmigene (PARallel Mutual Information calculation for GEneNEtwork reconstruction) available as a Bioconductor package. The optimal mutual information threshold was determined with a permutation test among 1000 runs where samples were randomly permuted and the inferred network was then pruned with Data Processing Inequality (DPI) analysis in order to limit the presence of indirect relationships.Their specific regulons were then imported into the IPA software (Ingenuity System Inc; <http://www.ingenuity.com/>) to identify the most enriched canonical pathways, over-represented biological processes and molecular functions associated to candidate genes. In order to confirm the role of CD15/FUT4 in specific subtype in additional data sets, we analyze the colorectal Cancer Genome Atlas (TCGA) series, Nature 2012, composed of 210 patients. This data set was added because of the extensive DNA alteration annotations and 20 normal colonic mucosa data provided (Cancer Genome Atlas Network, 2012). From such a dataset, we also examined the relationship between patients’ outcomes, where available, and *CD15/FUT4* transcript in terms of overall survival. Genome-wide transcription profile CD15/FUT4-correlated genes was estimated through positive or negative Pearson’s coefficient by using a stringent cutoff (at least 0.4 or -0.4). Gene Ontology (GO) pathway enrichment analysis of the resulting differentially expressed genes was conducted with Database for Annotation, Visualization and Integrated Discovery (DAVID)(http://david.abcc.ncifcrf.gov), while their functional characterization to identify possible enriched molecular networks and canonical pathways was performed using a proprietary software, Ingenuity Pathway Analysis (IPA) from Ingenuity Systems® (<http://www.ingenuity.com>). Statistical analyses were performed by GeneSpring R/bioconductor v.12.5 and R based package.

**Combination of CRC cell line data sets.**

To obtain a robust molecular classification of CD15/FTU4, we used a large set of 60 colon cancer cell lines from the “Cancer Cell Line Encyclopedia from the Broad Institute and Novartis, *Nature* 2012” and querying for annotated genomic profiles (mutations, putative copy-number alteration and mRNA expression microarrays) [8]. By clustering algorithm based on mutational loading and chromosomal instability (CIN) profiles, we determined that *CD15/FUT4*overexpression correlated with increased ERBB2, ERBB3 and FGFR4 transcript levels. By this approach, we did not find relationship between CD15/FUT4expression levels and copy number alterations (CNAs) or amplifications at the *CD15/FUT4* locus. We used a fold change of 1.5 (defined above) to assign differentially expressed genes (DEGs). CRC cell lines were defined as CIN+ (positive) or CIN- (negative) based on weighted genome instability index >0.2 (fraction of genome altered) [8-10]. Microarray data of metastatic CRC cell line SW480 displaying primary resistance to cetuximab, mutations in RAS/RAF/MEK/MAPK pathway and treated with the MEK inhibitor (AZD6244, Selumetinib) were obtained from GEO Omnibus and were analyzed using an unbiased screening approach [10-12]. The multiarray average preprocessing and normalization of raw CEL files from AffymetrixGeneChip® arrays were performed using R Bioconductor. Median centering of genes and clustering of samples and/or genes from the microarray data sets were performed using Gene Cluster 3.0. The clustering results were viewed using GenePattern based Hierarchical Clustering Viewer.

**Chemicals, reagents,** immunofluorescent **and western-blot analysis**

To determine whether *CD15/FUT4* expression is regulated by MAPK-ERK, we evaluated the response to pro-mitogenic and pro-inflammatory inductors EGF, IL1b, IL10, IL6 following kinase activation and transcript induction of target genes through kinase assays and qRT-PCR. Representative CRC cells were then grown to 70% of confluence, serum starved for 24h, and stimulated for 8h with 10 nM EGF (R&D System), 20U/ml IL-1beta (Peprotech). To assess the role of others factors on *CD15/FUT4* transcript, recombinant IL-10: 200U/ml (R&D System) IL-6: 50ng/ml were added for 30 minutes and subsequently the cells were harvested for RNA (qRT-PCR see above) or protein extraction. Western blot was performed according to the published procedures by using antibodies against: ERK 1/2 (MK1) and anti-p-ERK (E-4), dilution 1:100, Santa Cruz Biotechnology) (STAT1 total and Y701-STAT1, p-STAT1, dilution 1:500, Cell Signaling Technology), (STAT3 total, Y705-STAT3, p-STAT3, dilution 1:500, Cell Signaling Technology), (β-actin, dilution 1:10000, Sigma Aldrich was used as loading control) [2, 12]. A ratio of normalized ERK1/2 (pERK/total ERK1/2), Stat3 (pStat3/total Stat3**)** and stat1 (pstat1/total Stat1) was calculated for monitoring expression and phosphorylationlevels. In all experiments (0.1% DMSO indicated as vehicle) was used as negative control. Human polymorphonuclear cells (PMN) and peripheral blood mononuclear cells (PBMC) purified from buffy coats of healthy donors were used as positive control for STAT3 and STAT1 kinase assays [12]. For cell surface immunofluorescent analysis, RKO and HT29 cells were cultured on coverslips until 70% confluent. Next, they were fixed with 100% methanol for 15 min, washed with PBS and incubated with anti-CD15-FITC (clone MMA, Becton Dickinson, San Jose, CA), for 60 min. Nuclear co-stain was performed by using 4’6-diamidino-2-phenylindole (Dapi) for 1 min.

**Statistical analysis**

Prognostic and predictive effects were assessed using two clinical endpoints (PFS and OS).Kaplan–Meier plots were displayed and survival curves compared using log-rank test analysis.Prognostic analysis was performed in allpatients by using simple Cox proportional hazards model includinggene expression, gene mutation, and inflammatory response on PFS and OS. The Cox model was used to estimate hazard ratios (HRs) with 95% CIs for first-line treatment received Cetuximab or Bevacizumabplus chemotherapy based schedules (FOLFOX, XELOX or FOLFIRI). Multivariate models were adopted adjusting for factors known to be prognostic in mCRC (KRAS mutations, mucinous histology and therapeutic regime) or for baseline covariates (sex, age, ECOG, number of metastases, liver-only metastases).Receiver operating characteristic (ROC) curveswere applied to calculate the optimal cutoff point for NLR prognostic prediction. Groups were compared by analysis of variance using non-parametric Wilcoxon-Mann-Whitney and Kruskal-Wallis tests with median differences at 95% confidence interval (CI).The Spearman rank test was used to assess the correlation between continuous variables, and the Pearson X^2^ test for the association between categorical variables. Data are presented with medians and ranges. The *P* values were calculated two sided and considered as significant when ≤0.05. Statistical analyses were conducted by using R statistical software and SPSS (version 15 Windows, SPSS Inc, Chicago, IL), and GraphPad Prism 5.

**References**

1. Malietzis G, Giacometti M, Kennedy RH et al:[The emerging role of neutrophil to lymphocyte ratio in determining colorectal cancer treatment outcomes: a systematic review and meta-analysis.](http://www.ncbi.nlm.nih.gov/pubmed/24866438)Ann Surg Oncol 21(12):3938-46, 2014.
2. Pancione M, Remo A, Zanella C et al:[The chromatin remodelling component SMARCB1/INI1 influences the metastatic behavior of colorectal cancer through a gene signature mapping to chromosome 22.](http://www.ncbi.nlm.nih.gov/pubmed/24286138) J Transl Med 11:297S, 2013.
3. Pagnotta SM, Laudanna C, Pancione M et al:Ensemble of gene signatures identifies novel biomarkers in colorectal Cancer activated through PPAR𝛾 and TNF𝛼 signaling.PLoSOne8(8): e72638, 2013.
4. Cancer Genome Atlas Network. Comprehensive Molecular Characterization of Human Colon and Rectal Cancer.Nature 487: 330–37, 2012.
5. Smith JJ, Deane NG, Wu F et al: Experimentally derived metastasis gene expression profile predicts recurrence and death in patients with colon cancer. Gastroenterology 138: 958–968, 2010
6. Cerulo L, Elkan C, Ceccarelli M: Learning gene regulatory networks from only positive and unlabeled data. *BMC Bioinformatics* 2010,11:228.
7. Margolin Adam A et al: ARACNE: An Algorithm for the Reconstruction of Gene Regulatory Networks in a Mammalian Cellular Context. BMC Bioinformatics 2006, 7(Suppl 1):S7
8. Barretina J, Caponigro G, Stransky N et al: The Cancer Cell Line Encyclopedia enables predictive modelling of anticancer drug sensitivity. Nature 483(7391):603-7, 2012.
9. Sadanandam A, Lyssiotis CA, Homicsko Ket al:A colorectal cancer classification system that associates cellular phenotype and responses to therapy. Nature Med19(5): 619-25, 2013
10. [Schoumacher M](http://www.ncbi.nlm.nih.gov/pubmed/?term=Schoumacher%20M%5BAuthor%5D&cauthor=true&cauthor_uid=24747911), Hurov KE, Lehár et al: Inhibiting Tankyrases sensitizes KRAS-mutant cancer cells to MEK inhibitors via FGFR2 feedback signaling. Cancer Res 74(12): 3294-305, 2014.
11. Troiani T, Napolitano S, Vitagliano D et al:Primary and acquired resistance of colorectal cancer cells to anti-EGFR antibodies converge on MEK/ERK pathway activation and can be overcome by combined MEK/EGFR inhibition. Clin Cancer Res 20(14): 3775-86, 2014.
12. Curtale G, Mirolo M, Renzi TA et al:[Negative regulation of Toll-like receptor 4 signaling by IL-10-dependent microRNA-146b.](http://www.ncbi.nlm.nih.gov/pubmed/23798430)Proc Natl Acad Sci USA 110(28): 11499-504, 2013
