# Supplementary material for: Cancer-related CD15/FUT4 overexpression decreases benefit to agents targeting EGFR or VEGF acting as a novel RAF-MEK-ERK kinase downstream regulator in metastatic colorectal cancer
Source: J Exp Clin Cancer Res. 2015 Oct 1;34:108. doi: 10.1186/s13046-015-0225-7 (PMC4590269; doi:10.1186/s13046-015-0225-7)
Supplement: Additional file 3: — Supplementary Figures and legends. (PDF 767 kb) [file 13046_2015_225_MOESM3_ESM.pdf]

Supplementary Figure 1

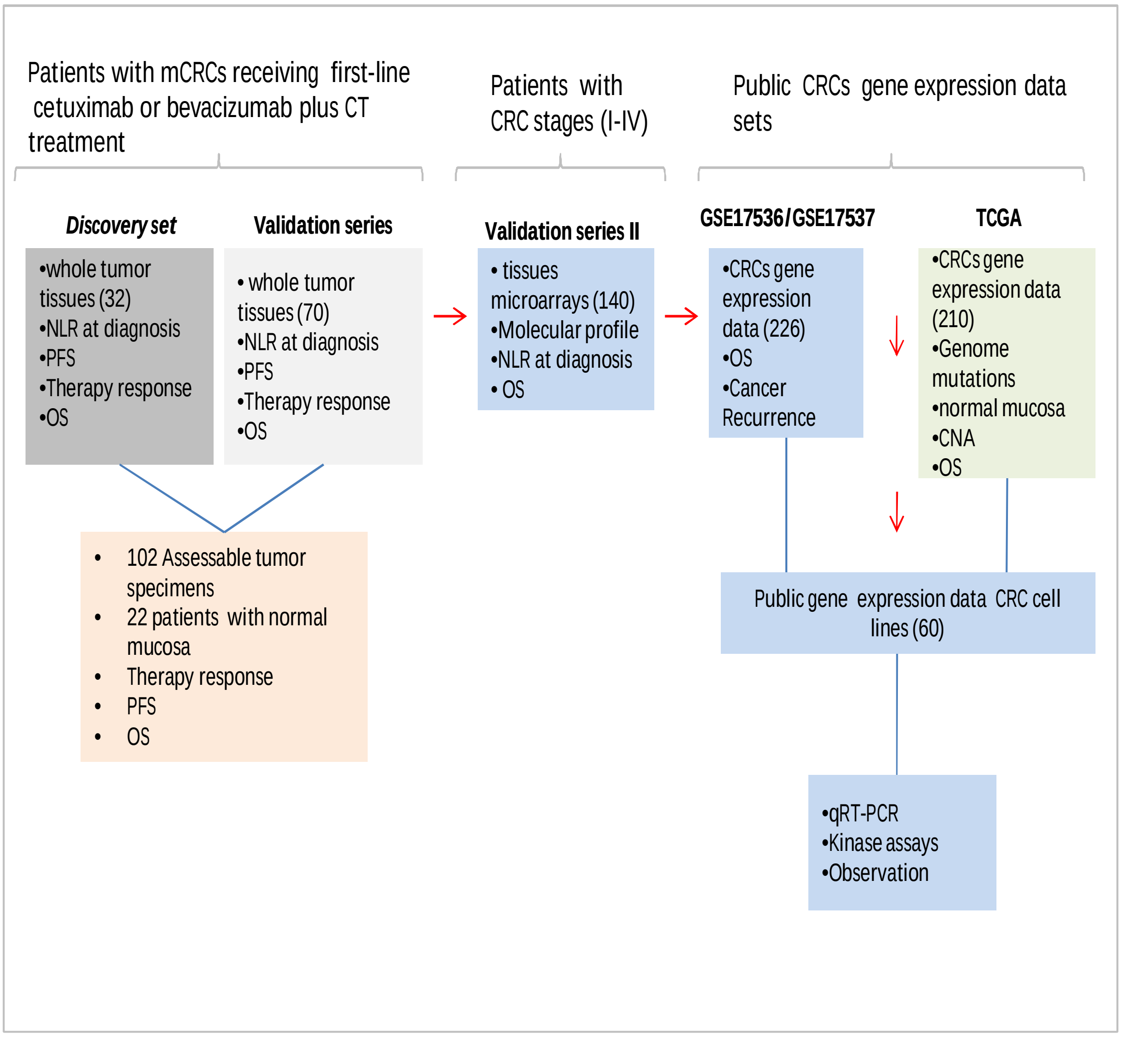

Supplementary Figure 1. Schematic overview of the study workflow is shown. Abbreviations: neutrophil-to-lymphocyte ratio, NLR; Progression-Free Survival, PFS; Overall Survival, OS; chemotherapy, CT; CNA, copy-number alterations.

Supplementary Figure 2

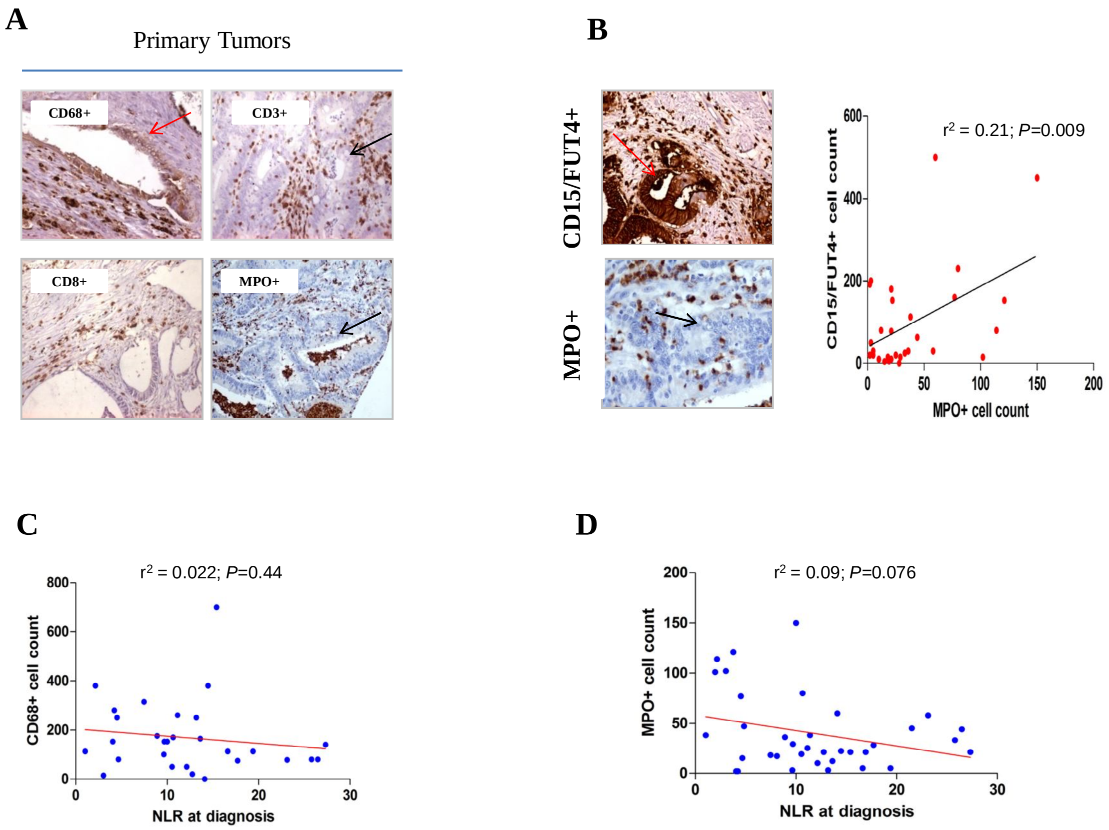

Supplementary Figure 2. Tumor-associated infiltrating immune cells and inflammatory response in discovery set of mCRC.

A)Representative images of immunostochemical determination of four inflammatory cell types pattern in CRC specimens. CD68 for monocyte-macrophage lineage cells, CD3 for T cells, CD8 for cytotoxic T cells and MPO for neutrophil granulocytes. Red and black arrows indicate presence or absence of immunohistochemical positivity on malignant colonic cells, respectively. **B)** Comparison between CD15/FUT4 and MPO immunostaining pattern where red and black arrows indicate presence or absence of positivity on malignant colonic cells, respectively. Correlation between tumor-associated CD15/FUT4 and MPO infiltrating neutrophil granulocytes indicates a high concordance (n=32). **C,D)** Correlation of NLR at diagnosis with quantification of $CD68^{+}$ and MPO^{+} tumor-associated macrophage and neutrophil granulocytes, respectively. **Abbreviations:** neutrophil-to-lymphocyte ratio, NLR; Myeloperoxidase, MPO.

Supplementary Figure 3

A

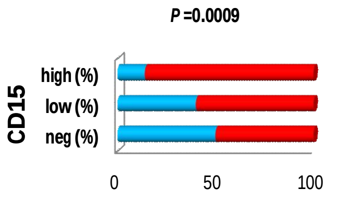

	neg (%)	low (%)	high (%)
NLR ≤5	50	40	14
NLR >5	50	60	86

C

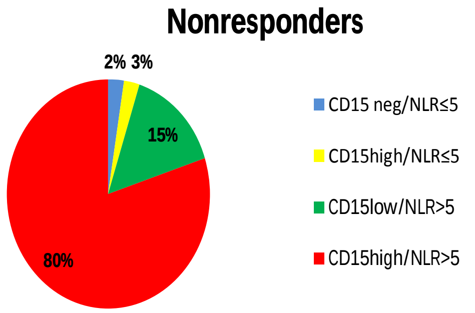

B

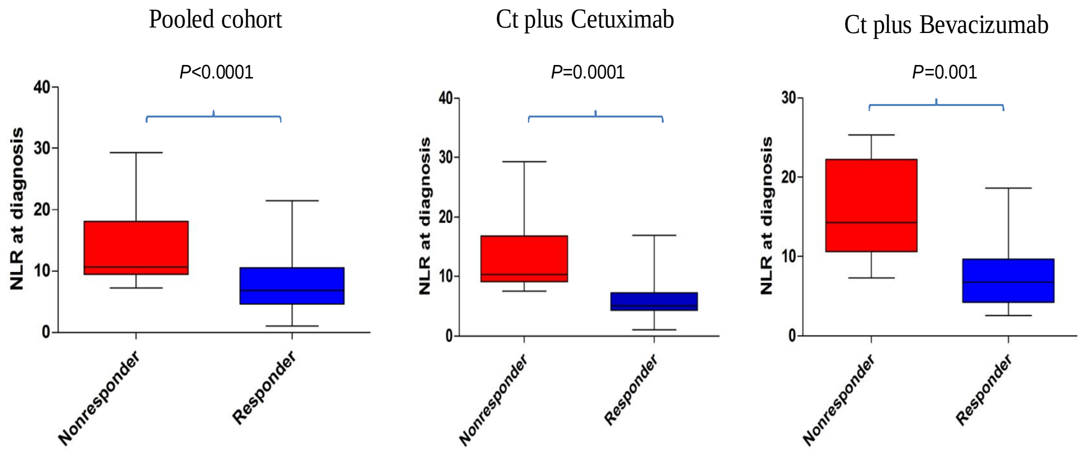

Supplementary Figure 3. CD15/FUT4 prognostic significance and inflammatory response in mCRCs.

A)Tumor-related CD15/FUT4 expression (IHC) according to NLR at diagnosis considering a cut off value (NLR >5 and NLR ≤5), respectively. The *P* value was obtained by chi-square test. **B)** Relationship between NLR at diagnosis "analyzed as a continuous variable" and response to treatments in the entire cohort or subdivided into subgroup treated with cetuximab (n=48) or bevacizumab (n=54) plus chemotherapy schedules, respectively. P value was obtained by Mann–Whitney test**. C)** Mutual relationship between CD15/FUT4 expression pattern and (NLR ≤5 or NLR >5) into subgroup of nonresponders (n=40). **Abbreviations:** neutrophil-to-lymphocyte ratio, Immunohistochemistry, IHC.

Supplementary Figure 4

A

CD15/FUT4 -TMAs validation series;

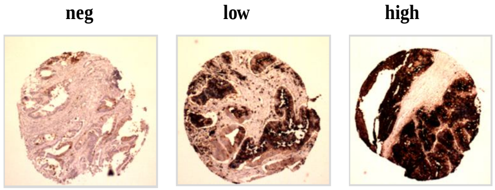

B

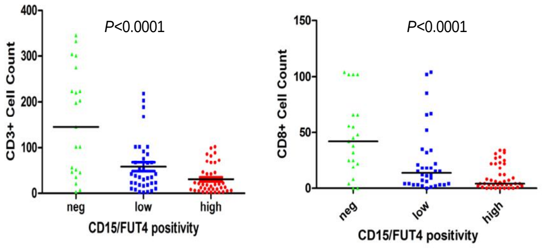

C

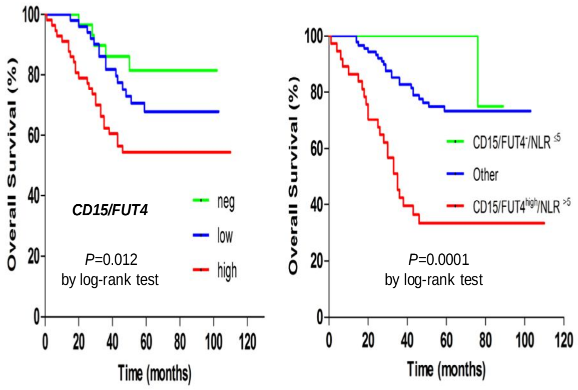

D

Univariate	HR (95%CI)	*P* value
CD15/FUT4 high vs low/neg	1.64 (1.09-2.45)	0.016
CD14/FUT4high/NLR>5	3.30 (1.81-6.02)	0.0001

Supplementary Figure 4. CD15/FUT4 (IHC) expression in the TMAs validation series comprising stage (I-IV) tumors

A)Representative images of CD15/FUT4 expression pattern in three cores of the TMAs validation series comprising (n=140) stage I-IV tumors. **B)** Relationship between CD15/FUT4 expression pattern and CD3 for T cells, CD8 for cytotoxic T cells infiltrating immune cells into TMAs data set. **C)** Kaplan–Meier curves for overall survival in the TMAs validation set (n=140) in relation with CD15/FUT4-high low and negative tumors and NLR at diagnosis (cut off 5). The likelihood-ratio test by univariate Cox-regression analysis confirmed that prognostic model for OS is improved by combining NLR >5 at diagnosis.

Supplementary Figure 5

A

CD15/FUT4 (REGULON)	N° of genes
HNRNPM	240
RPS6KB2	132
CDT1	125
INTS5	113
PDE12	100
MRPL16	64
E2F8	63
GEMIN4	61
ALYREF	56
ACTR3B	50
SURF1	48
RAD51	46
TMEM204	45
KIF2A	44
LINC00514	40
DHCR7	33
C11orf82	24
PPP1R14A	18
SETD9	15
PF4	13

B

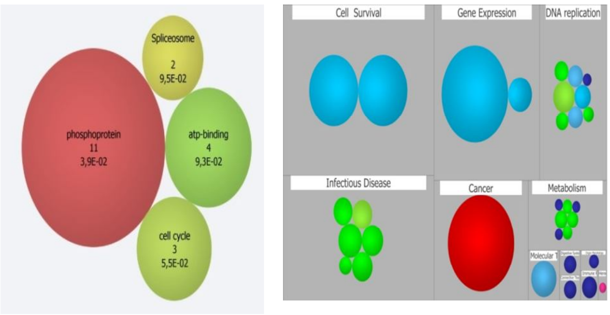

B

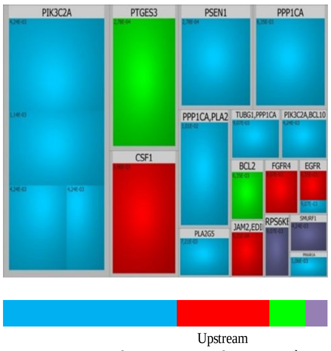

C

TCGA dataset (n=210)

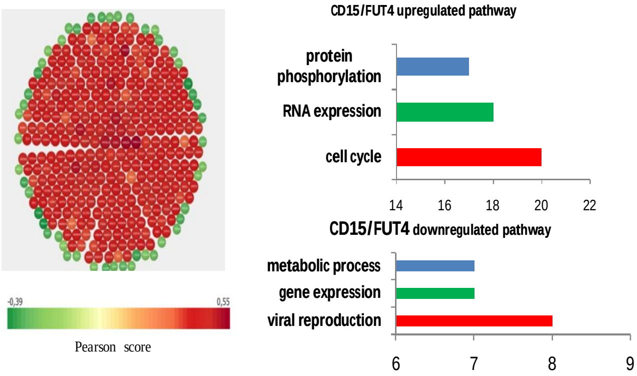

Supplementary Figure 5. Inferred CD15/FUT4 regulators across three independent genome-wide expression profiles

A)List of 20 most enriched regulons connected with CD15/FUT4 transcriptional network inferred by ARACNe algorithm from GSE17536/GSE17537 data sets. The size of each regulon, "group of genes under the control of a regulatory molecule" is indicated through different colors. The three most enriched regulons are indicated in red. **B)** Gene Ontology (GO) analysis "left" of the regulons showed significant enrichments for biological processes resulting in protein kinase cascade 11 out of 20 (55%) compared to others. A treemap representation of the most specific enriched biological processes is indicated. The size of the rounds and their recurrence is proportional to the significance of the enrichment. The same color indicates cluster of genes having overlapping biological functions. C) After removing the weakness interactions, a treemap analysis indicated that the top-ranking categories were related to genes involved in kinase cascade involving two main upstream regulators EGFR and FGFR pathways. D) Available gene expression data (GEP) were extracted from TCGA data set and analyzed for genome-wide transcription profile of the genes significantly correlated with *CD15/FUT4 mRNA* levels taking into account Pearson's score. Green indicates the genes negatively correlated and red those positively correlated to *CD15/FUT4* transcript. Gene Ontology (GO) analysis in this data set confirmed that the top-ranking categories were related on one hand to cell cycle, kinase cascade and on the other hand to the infectious disease.

Supplementary Figure 6

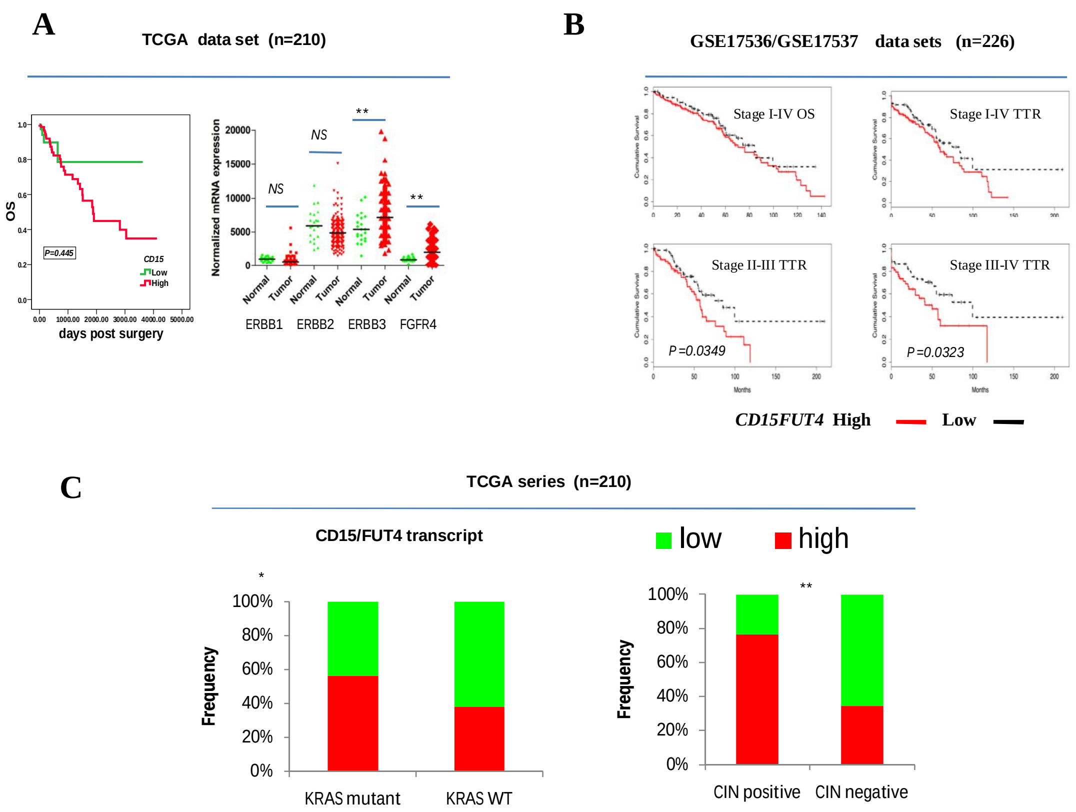

Supplementary Figure 6. Significance of CD15/FUT4, EGFRs and FGFR4 transcript levels and MEKi responses across independent gene-expression data sets.

A) Kaplan-Meier survival analysis is carried out taking into account *CD15/FUT4*-low (under the 25th percentile) and high (upper the 25th percentile**)** expressing tumors from TCGA data set. Comparison of *ERBB1, ERBB2, ERBB3 (EGFRs)* and *FGFR4* transcripts levels in CRC samples and normal intestinal epithelium derived from RNAseq transcriptomic data (TCGA data set). **B)** Kaplan-Meier overall survival and time-to-recurrence (TTR) analyses in relation to CD15/FUT4 transcript levels categorized as high and low (see above) in GSE17536/GSE17537 series. The P values were obtained by log-rank test (survival) or by Mann–Whitney test (expression)**;** $*P \leq .05$; $**P \leq .01$; non significant, NS. **C)** Relationship among the frequency of KRAS mutations, chromosomal instability and CD15/FUT4 transcript levels categorized as high and low from (TCGA data set). The *P* values were obtained by chi-square test. P value was obtained by Mann–Whitney test.
